# Association Between Mean Chewing Count per Bite During Rice Ball Consumption and the Postprandial Salivary Secretory Immunoglobulin a Response in Healthy Adult Men: A Pilot Study

**DOI:** 10.3390/dj14080519

**Published:** 2026-08-13

**Authors:** Koichiro Tabata, Tetsuji Azuma, Komei Iwai, Takatoshi Yonenaga, Gen Tanabe, Takaaki Tomofuji

**Affiliations:** 1 Department of Community Oral Health, School of Dentistry, Asahi University, Mizuho 501-0296, Japan; tabata-k@asahi-u.ac.jp (K.T.); tetsuji@asahi-u.ac.jp (T.A.); ko-mei@asahi-u.ac.jp (K.I.); yone0730@asahi-u.ac.jp (T.Y.); 2 Department of Sports Dentistry, School of Dentistry, Meikai University, Sakado 350-0283, Japan; gen.spmd@dent.meikai.ac.jp

**Keywords:** chewing behavior, mastication, salivary IgA, IgA secretion rate, saliva

## Abstract

**Background/Objectives:** Mastication may influence oral mucosal immunity; however, its association with postprandial salivary secretory immunoglobulin A (s-IgA) dynamics remains unclear. This pilot study examined the association between mean chewing count per bite during rice ball consumption and the postprandial salivary s-IgA response in healthy men. **Methods:** Thirty-seven healthy adult men were enrolled. Mean chewing count per bite was analyzed continuously and secondarily in exploratory groups defined by the median (<30, *n* = 18; ≥30 chews/bite, *n* = 19). Unstimulated whole saliva was collected before, 0–5, 10–15, and 25–30 min after eating. Salivary s-IgA concentration, saliva weight, and s-IgA secretion rate were evaluated as absolute values and baseline ratios. Baseline-adjusted, repeated-measures, and sensitivity analyses were performed. **Results:** Mean chewing count per bite correlated positively with normalized salivary s-IgA concentration at 10–15 min (ρ = 0.347, *p* = 0.036) and 25–30 min (ρ = 0.352, *p* = 0.033), but these associations were attenuated after false discovery rate correction (q = 0.053 for both). Baseline salivary s-IgA was inversely associated with normalized responses. In exploratory group comparisons, normalized salivary s-IgA concentration was higher in the ≥30 chews/bite group at 10–15 and 25–30 min, whereas absolute s-IgA concentration, saliva weight, and s-IgA secretion rate did not differ between groups. Baseline-adjusted associations were not statistically significant. **Conclusions:** Higher mean chewing count per bite may be associated with greater relative postprandial salivary s-IgA responses in healthy adult men, but these findings are exploratory and should be interpreted cautiously.

## 1. Introduction

Oral function is not limited to food intake and speech, but rather, is increasingly recognized as being related to systemic health [1,2]. Among oral functions, mastication plays a central role in food comminution and swallowing and may have broader physiological relevance beyond food processing, with possible links to neural, endocrine, and immune regulation [3,4,5].

Saliva is a multifunctional biological fluid that contributes to oral homeostasis, lubrication, taste perception, swallowing, and the initial stages of digestion [6]. Secretory immunoglobulin A (s-IgA), the predominant immunoglobulin in saliva, is a major component of mucosal immunity and serves as a first-line defense against bacteria, viruses, and other external antigens [7,8,9,10]. By limiting microbial adhesion and penetration at mucosal surfaces without provoking marked inflammation, s-IgA contributes to immune exclusion and the maintenance of oral homeostasis [8,9,10].

Previous studies have shown that chewing stimulation can enhance salivary secretion and increase salivary s-IgA output [11,12]. However, salivary responses during eating are affected by not only the presence of mastication itself, but also oral mechanical stimulation, taste, texture, and the physical properties of food [6,13,14]. These observations suggest that chewing quality and patterns may influence postprandial salivary immune responses.

A rice ball provides a standardized and ecologically relevant test food for evaluating mastication under familiar eating conditions in Japan [15]. In addition, a wearable chewing sensor has enabled objective quantification of chewing behavior during actual food consumption [15,16]. Nevertheless, the relationship between the mean chewing count per bite and postprandial salivary s-IgA dynamics remains insufficiently understood. Therefore, the present pilot study aimed to examine the association between the mean chewing count per bite during rice ball consumption and the postprandial salivary s-IgA response in healthy adult men.

## 2. Materials and Methods

### 2.1. Study Design and Participants

Participants were recruited from among students at Asahi University in Gifu Prefecture, Japan. The analysis included 37 healthy young adult men. Because this was an exploratory pilot study, recruitment was restricted to men to reduce biological variability and to establish initial data before future studies including women. No formal sample size calculation was performed because of the exploratory nature of the study.

Eligible participants had natural dentition excluding third molars and showed no subjective or objective abnormalities in oral function. Objective oral health assessments included the number of present teeth, decayed, missing, and filled (DMF) teeth, Community Periodontal Index (CPI) scores for periodontal pockets and gingival bleeding [17], and the Oral Hygiene Index (OHI) [18]. All participants were non-smokers. Exclusion criteria were current use of medications that could affect study outcomes, including antimicrobial agents, ongoing dental treatment, body mass index ≥30 kg/m^2^, food allergies, and a history of psychiatric disorders.

This study was conducted in accordance with the Declaration of Helsinki. The study protocol was approved by the Ethics Committee of the School of Dentistry, Asahi University (approval No. 36006; approval date: 20 July 2024) and was registered with the Japan Registry of Clinical Trials (jRCT1040260006) on 13 April 2026, before participant recruitment. Participants were recruited between 14 April 2026 and 30 April 2026. Written informed consent was obtained from all participants before enrollment.

### 2.2. Experimental Procedure and Saliva Collection

To minimize the influence of diurnal variation in salivary s-IgA concentrations, all examinations were conducted between 16:30 and 18:30 [19]. Participants were instructed to refrain from eating, drinking anything other than water, and brushing their teeth for at least 1 h before saliva collection. Each participant consumed one 100 g salted rice ball (Kanemi Foods Co., Ltd., Hashima, Japan), and chewing behavior during consumption was recorded.

Unstimulated whole saliva was collected by passive drooling for 5 min at four time points: before eating, immediately after eating (0–5 min), 10–15 min after eating, and 25–30 min after eating. Saliva samples were used to determine salivary s-IgA concentration, saliva weight, and s-IgA secretion rate.

### 2.3. Assessment of Chewing Behavior

Chewing behavior was measured using a wearable chewing sensor (bitescan BH-BS1RR; Sharp Co., Ltd., Osaka, Japan), which has previously been used for the objective assessment of masticatory behavior [15,16]. The device was connected via Bluetooth to a smartphone (SH-M15; Sharp Co., Ltd.), and data were recorded using the dedicated application (version 1.7.04).

To maintain procedural consistency, the bitescan device was placed on the right ear of all participants, irrespective of individual masticatory laterality. During device placement, participants were seated in a dental chair with the Frankfurt plane parallel to the floor and instructed to maintain a natural head position.

The following chewing-related variables were recorded during rice ball consumption: total eating time (in seconds), total chewing count, chewing tempo (chews/min), and mean chewing count per bite. Mean chewing count per bite was calculated by dividing total chewing count by the number of bites detected by the bitescan device.

### 2.4. Measurement of Salivary s-IgA and Calculation of s-IgA Secretion Rate

Salivary s-IgA concentration was measured using a sandwich immunoassay with antibody-labeled gold nanoparticles (ImmunoSense Co., Ltd., Osaka, Japan) [20]. The lower limit of detection of this method was approximately 1 pg/mL.

Saliva weight was used as a proxy for saliva volume, assuming that 1 g of saliva was equivalent to 1 mL. The salivary s-IgA secretion rate (µg/min) was calculated by multiplying salivary s-IgA concentration (µg/mL) by saliva volume collected during each 5 min sampling period and dividing the obtained value by 5 to express the result per minute. For relative analyses, postprandial saliva weight, salivary s-IgA concentration, and salivary s-IgA secretion rate were expressed as ratios relative to the pre-chewing value, with the pre-chewing value set at 100 for each participant.

### 2.5. Outcome Measures

The primary exposure variable was mean chewing count per bite during rice ball consumption. The primary analysis treated mean chewing count per bite as a continuous exposure variable. For secondary exploratory descriptive analyses, participants were also classified into two groups according to mean chewing count per bite using a cut-off of 30 chews/bite, which corresponded to the sample median: <30 and ≥30 chews/bite. Although a similar chewing-count condition has been used in previous physiological research [21], this cut-off was used to create two groups of comparable size for exploratory comparison and should not be interpreted as an established biological threshold.

Study outcomes included salivary s-IgA concentration, saliva weight, and salivary s-IgA secretion rate at each time point, together with their postprandial ratios relative to the pre-chewing value. Particular attention was paid to pre-chewing-normalized salivary s-IgA concentration as an exploratory response indicator; however, salivary s-IgA secretion rate was also considered important because it reflects the amount of s-IgA delivered to the oral cavity while accounting for salivary flow.

### 2.6. Statistical Analysis

Data are presented as median and interquartile range (IQR) for continuous variables and as number and percentage for categorical variables. Between-group comparisons of continuous variables were performed using the Mann–Whitney U test. CPI periodontal pocket and bleeding scores were dichotomized as absent (score 0) or present (score ≥ 1), and between-group comparisons were performed using Fisher’s exact test. To reduce the effect of interindividual variability, postprandial changes in salivary s-IgA concentration, saliva weight, and s-IgA secretion rate were also expressed as ratios relative to the pre-chewing value, with the pre-chewing value set at 100.

Because all postprandial values were expressed relative to the pre-chewing value (defined as 100), Dunnett-adjusted comparisons were used to determine whether each postprandial time point differed significantly from the common reference value while controlling for multiple testing. These within-group analyses were performed to assess temporal change relative to baseline within each chewing group and were not intended to provide evidence of between-group differences. The association between mean chewing count per bite as a continuous variable and pre-chewing-normalized salivary s-IgA concentration was assessed using Spearman’s rank correlation coefficient. To account for multiplicity across the three postprandial time points, the Benjamini–Hochberg false discovery rate (FDR) procedure was applied within each family of Spearman correlation analyses (the primary analysis and each sensitivity-analysis set), but was not applied to the baseline-adjusted regression analyses or the GEE analyses. Because these models estimated prespecified regression coefficients, each coefficient is reported with its 95% confidence interval. To account for baseline differences among participants, baseline-adjusted analyses were performed using linear regression models with log-transformed postprandial salivary s-IgA concentration as the dependent variable, mean chewing count per bite as the independent variable, and log-transformed baseline salivary s-IgA concentration as a covariate. To account for the repeated-measures structure of the data, GEE with an exchangeable working correlation structure and robust (sandwich) standard errors were additionally fitted, including postprandial time point as a repeated within-subject factor, mean chewing count per bite as a continuous predictor, and their two-way interaction. An autoregressive AR(1) working correlation structure was fitted as a sensitivity analysis to assess the robustness of the GEE results. Sensitivity analyses were also performed after excluding participants with mean chewing counts exceeding 100 chews/bite and after excluding the statistical outlier identified using Tukey’s rule. The prespecified analytical hierarchy was as follows: (i) Spearman correlations between mean chewing count per bite and pre-chewing-normalized salivary s-IgA concentration as the primary analysis; (ii) baseline-adjusted linear regression analyses evaluating the same association; (iii) GEE repeated-measures analyses; and (iv) sensitivity analyses. The secondary group comparison (<30 vs. ≥30 chews/bite, sample-median split) is reported only for descriptive purposes. All between-group statistical tests were two-sided, and *p* values < 0.05 were considered statistically significant. Statistical analyses were performed using SPSS Statistics version 27 (IBM Japan, Tokyo, Japan).

## 3. Results

### 3.1. Participants’ Characteristics

A total of 37 healthy adult men were included in the analysis. Across all participants, the median [IQR] mean chewing count per bite was 30.0 [20.0–74.0]. Participants were classified into two groups for secondary exploratory descriptive analyses: <30 chews/bite (*n* = 18) and ≥30 chews/bite (*n* = 19). No adverse events or unintended effects were observed during the study.

Pre-chewing data are summarized in Table 1. Age did not differ significantly between the two groups (<30 chews/bite: 23.0 [22.2–25.5] years; ≥30 chews/bite: 25.0 [22.0–28.5] years; *p* = 0.350). Eating time was also comparable between groups (194.5 [162.0–263.0] s vs. 194.0 [170.0–230.0] s; *p* = 0.939). Total chewing count tended to be higher in the ≥30 chews/bite group, although this difference did not reach statistical significance (237.5 [161.2–283.5] vs. 285.0 [252.5–343.0]; *p* = 0.052). As expected, chewing tempo and mean chewing count per bite were significantly higher in the ≥30 chews/bite group (chewing tempo: 72.5 [64.5–79.0] chews/min vs. 88.0 [83.5–92.5] chews/min, *p* < 0.001; mean chews per bite: 20.0 [14.5–26.0] vs. 74.0 [44.0–121.0], *p* < 0.001). Baseline saliva weight, salivary s-IgA concentration, salivary s-IgA secretion rate, and objective oral health indices did not differ significantly between groups.

### 3.2. Continuous Analysis of Mean Chewing Count per Bite and Postprandial Salivary s-IgA Response

**Figure 1:**
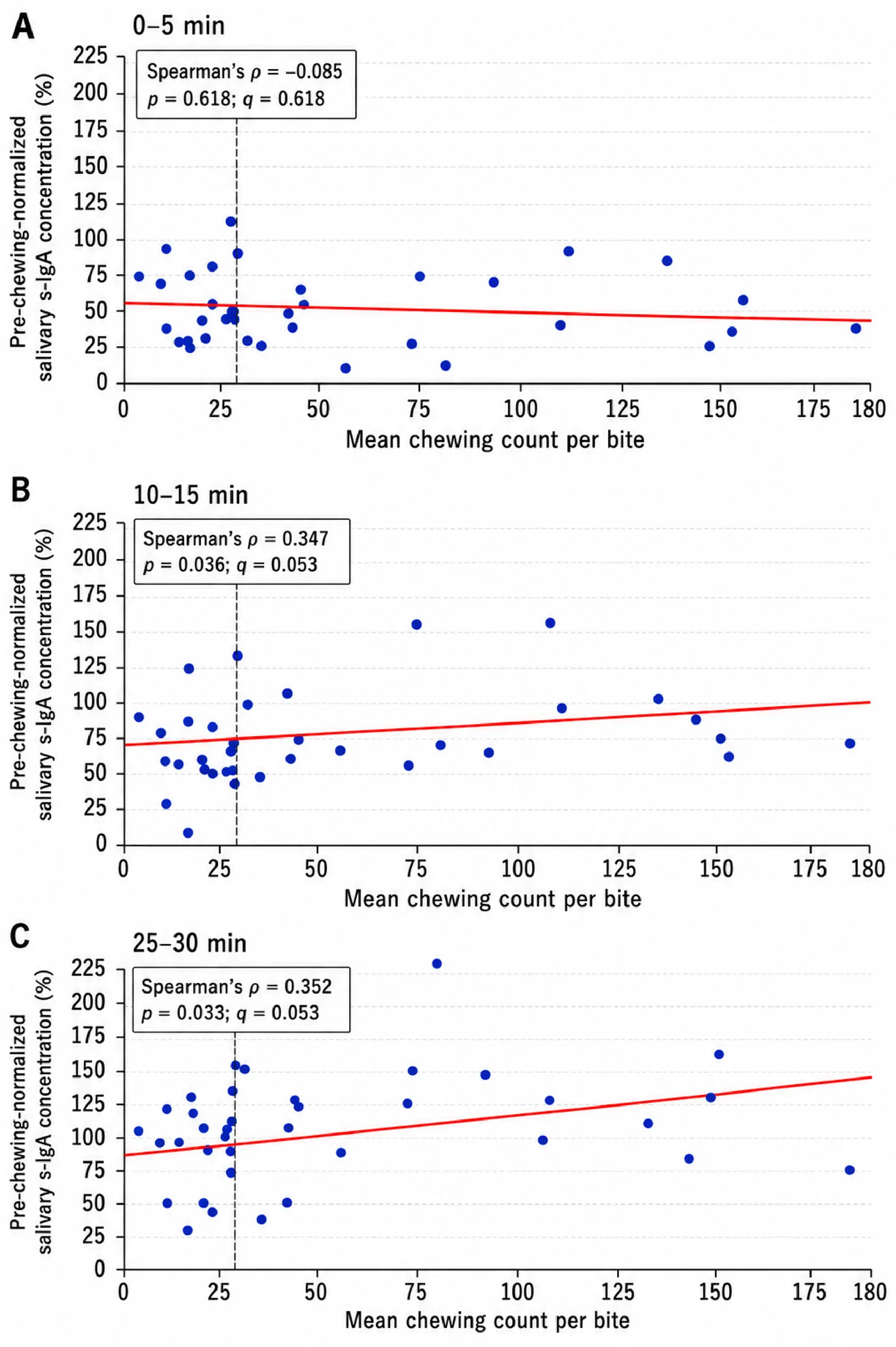
Association between mean chewing count per bite and pre-chewing-normalized salivary s-IgA concentration after rice ball consumption. Scatterplots show the relationship between mean chewing count per bite and pre-chewing-normalized salivary secretory immunoglobulin A (s-IgA) concentration at 0–5 (**A**), 10–15 (**B**), and 25–30 min (**C**) after eating. The dashed vertical line indicates the sample-median cut-off of 30 chews/bite used for the secondary exploratory group comparison. The red solid line indicates the linear regression line. Associations were evaluated using Spearman’s rank correlation coefficient. No significant association was observed at 0–5 min, whereas nominally positive correlations were observed at 10–15 min (ρ = 0.347, *p* = 0.036, q = 0.053) and 25–30 min (ρ = 0.352, *p* = 0.033, q = 0.053).

Spearman correlation analysis showed no significant association between mean chewing count per bite and pre-chewing-normalized salivary s-IgA concentration at 0–5 min (ρ = −0.085, *p* = 0.618), whereas nominal positive associations were observed at 10–15 min (ρ = 0.347, *p* = 0.036) and 25–30 min (ρ = 0.352, *p* = 0.033) after eating (Figure 1). However, after FDR correction across the three postprandial time points, these associations were attenuated (q = 0.053 for both 10–15 and 25–30 min).

Baseline salivary s-IgA concentration was inversely associated with pre-chewing-normalized postprandial salivary s-IgA concentration at 0–5 min (ρ = −0.374, *p* = 0.023) and 10–15 min (ρ = −0.337, *p* = 0.041), with a weaker tendency at 25–30 min (ρ = −0.278, *p* = 0.095), indicating that ratio normalization did not fully eliminate baseline-related variation.

In baseline-adjusted analyses using log-transformed postprandial salivary s-IgA concentration, the association between mean chewing count per bite and postprandial salivary s-IgA concentration became less pronounced and was not statistically significant at any time point (0–5 min: β = −0.0010, 95% CI −0.0048 to 0.0028, *p* = 0.597; 10–15 min: β = 0.0028, 95% CI −0.0014 to 0.0070, *p* = 0.184; 25–30 min: β = 0.0032, 95% CI −0.0017 to 0.0081, *p* = 0.192).

### 3.3. Secondary Exploratory Group Comparison of Postprandial Salivary Responses

Absolute salivary s-IgA concentration did not differ significantly between the <30 and ≥30 chews/bite groups at any time point (Table 2). However, when postprandial changes were expressed relative to the pre-chewing value, pre-chewing-normalized salivary s-IgA concentration was significantly higher in the ≥30 chews/bite group at 10–15 and 25–30 min after eating. At 10–15 min after eating, the pre-chewing-normalized salivary s-IgA concentration was 56.2 [49.5–73.5] in the <30 chews/bite group and 74.0 [64.3–102.3] in the ≥30 chews/bite group (*p* = 0.005). At 25–30 min after eating, the corresponding values were 91.2 [57.0–104.5] and 118.5 [90.1–139.5], respectively (*p* = 0.022). No significant between-group difference was observed at 0–5 min after eating (44.3 [37.1–70.0] vs. 39.6 [28.4–65.2]; *p* = 0.457). Absolute saliva weight did not differ significantly between groups at any time point, and no significant between-group differences were observed in pre-chewing-normalized saliva-weight ratios at any postprandial time point. The absolute salivary s-IgA secretion rate did not differ significantly between the <30 and ≥30 chews/bite groups at any time point. The pre-chewing-normalized s-IgA secretion rate also showed no significant between-group difference at any postprandial time point.

### 3.4. Within-Group Comparison of Postprandial Salivary s-IgA Response

**Figure 2:**
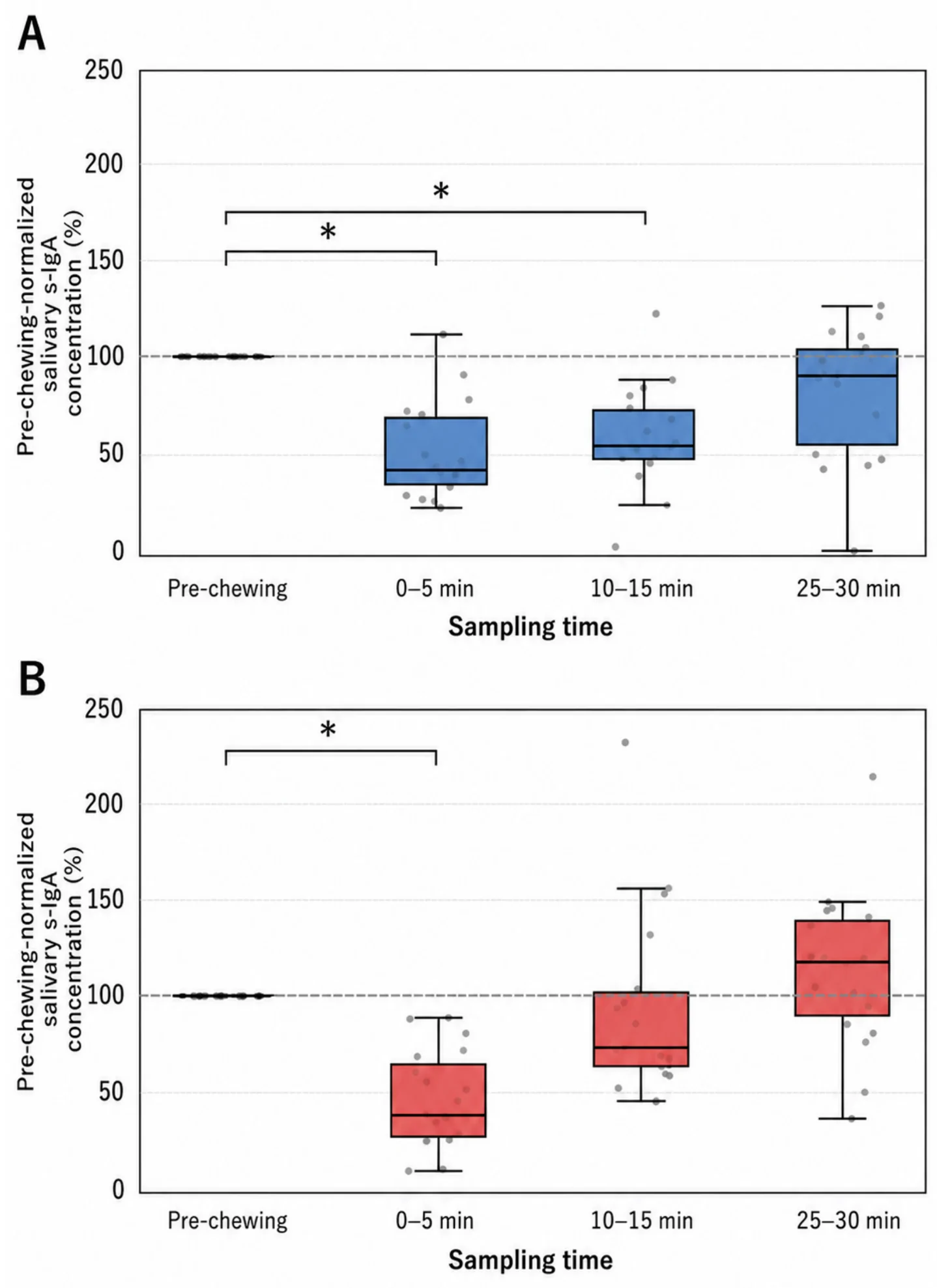
Pre-chewing-normalized salivary s-IgA concentration according to chewing frequency. Box-and-whisker plots show the pre-chewing-normalized salivary s-IgA concentration in the <30 (**A**) and ≥30 chews/bite groups (**B**) at pre-chewing, 0–5, 10–15, and 25–30 min after rice ball consumption. Boxes indicate the median and interquartile range; whiskers indicate the range excluding outliers; gray dots indicate individual participants. The dashed horizontal line indicates the pre-chewing reference value of 100. Within-group comparisons were performed using Dunnett-adjusted comparisons. Significant differences versus pre-chewing are indicated by brackets. * *p* < 0.05 versus pre-chewing.

Within-group analysis using Dunnett adjustment for multiple comparisons indicated that the pre-chewing-normalized salivary s-IgA concentration was significantly lower than the pre-chewing value at 0–5 and 10–15 min in the <30 chews/bite group, and at 0–5 min in the ≥30 chews/bite group (Figure 2).

**Figure 3:**
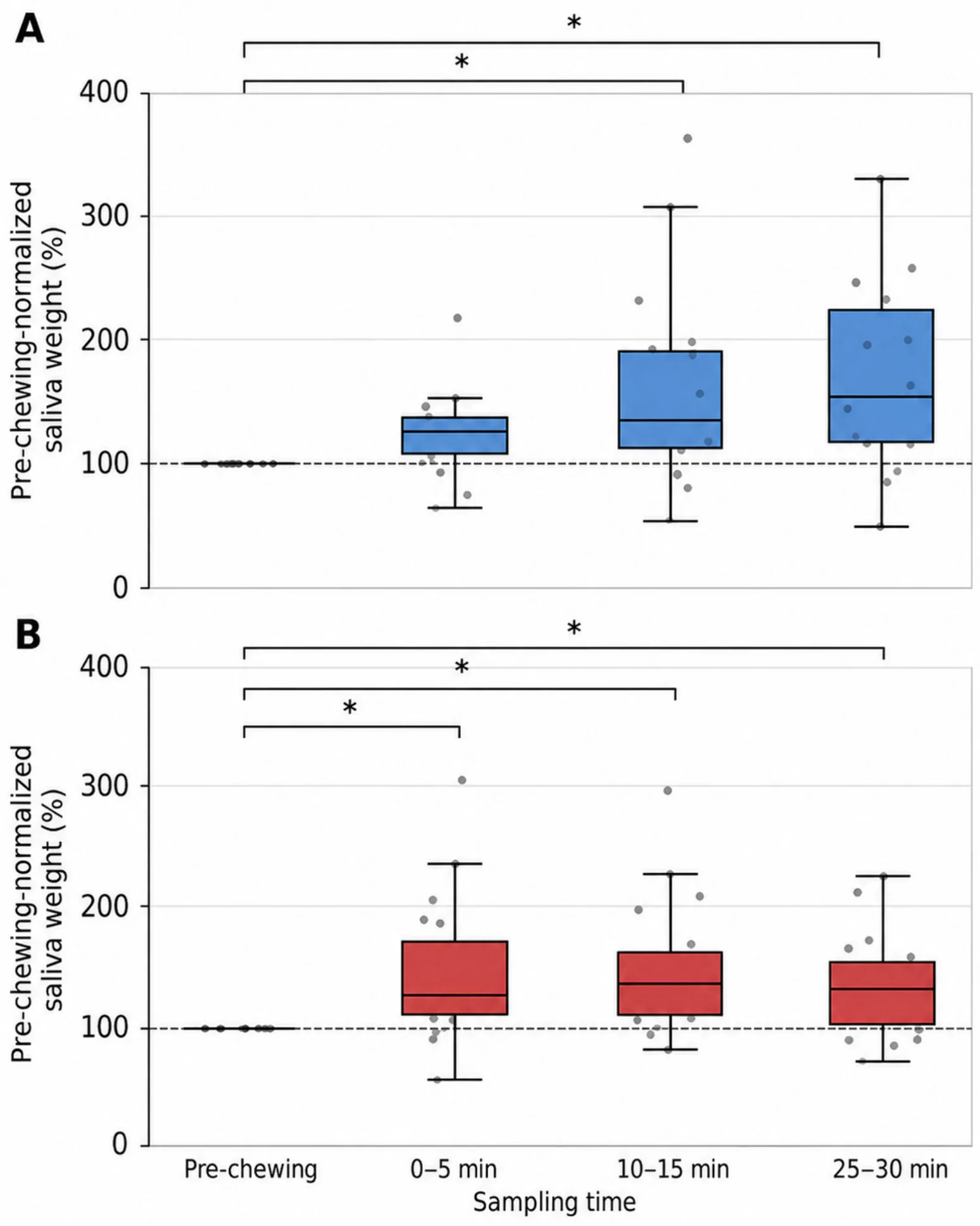
Pre-chewing-normalized saliva weight according to chewing frequency. Box-and-whisker plots show pre-chewing-normalized saliva weight in the <30 (**A**) and ≥30 chews/bite groups (**B**) at pre-chewing, 0–5, 10–15, and 25–30 min after rice ball consumption. Boxes indicate the median and interquartile range; whiskers indicate the range excluding outliers; gray dots indicate individual participants. The dashed horizontal line indicates the pre-chewing reference value of 100. Within-group comparisons were performed using Dunnett-adjusted comparisons. Significant differences versus pre-chewing are indicated by brackets. * *p* < 0.05 versus pre-chewing.

By contrast, pre-chewing-normalized saliva-weight ratios increased significantly from the pre-chewing value at all postprandial time points in both groups (Figure 3).

**Figure 4:**
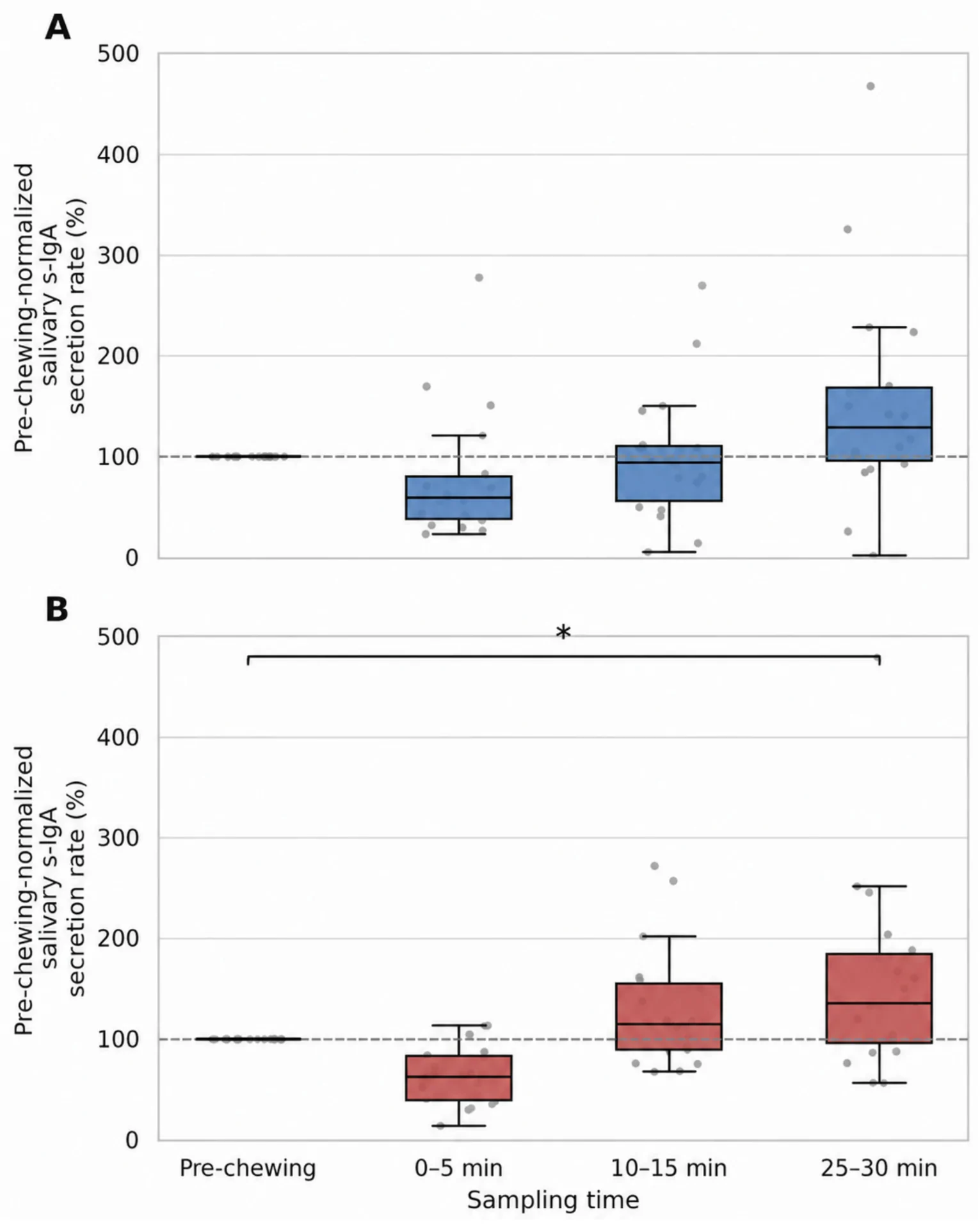
Pre-chewing-normalized salivary s-IgA secretion rate according to chewing frequency. Box-and-whisker plots show the pre-chewing-normalized salivary s-IgA secretion rate in the <30 (**A**) and ≥30 chews/bite groups (**B**) at pre-chewing, 0–5, 10–15, and 25–30 min after rice ball consumption. Boxes indicate the median and interquartile range; whiskers indicate the range excluding outliers; gray dots indicate individual participants. The dashed horizontal line indicates the pre-chewing reference value of 100. Within-group comparisons were performed using Dunnett-adjusted comparisons. Significant differences versus pre-chewing are indicated by brackets. * *p* < 0.05 versus pre-chewing.

Furthermore, pre-chewing-normalized s-IgA secretion rate was significantly increased at 25–30 min only in the ≥30 chews/bite group, whereas no significant increase was observed in the <30 chews/bite group (Figure 4).

### 3.5. Repeated-Measures and Sensitivity Analyses

In the GEE analysis of pre-chewing-normalized salivary s-IgA concentration, the interaction terms between mean chewing count per bite and postprandial time were β = 0.189 (95% CI −0.018 to 0.396, *p* = 0.074) at 10–15 min and β = 0.259 (95% CI 0.021 to 0.498, *p* = 0.033) at 25–30 min, whereas the joint Wald test for the two interaction terms was borderline (Wald χ^2^ = 5.718, d.f. = 2, *p* = 0.057). Sensitivity analyses were performed after excluding participants with mean chewing counts exceeding 100 chews/bite and after excluding the Tukey-defined outlier. In both analyses, the overall direction of association between mean chewing count per bite and pre-chewing-normalized salivary s-IgA concentration remained similar, although the statistical strength varied modestly depending on the exclusion rule (Supplementary Table S1).

## 4. Discussion

In this exploratory pilot study of healthy young adult men, greater mean chewing count per bite was associated with higher pre-chewing-normalized postprandial salivary s-IgA concentrations at 10–15 and 25–30 min after rice ball consumption in nominal analyses. However, these associations were attenuated after false discovery rate correction and became less pronounced after baseline adjustment. In addition, no significant between-group differences were observed in salivary s-IgA secretion rate at any postprandial time point. Therefore, the present findings should be interpreted as exploratory evidence of an association rather than definitive evidence that greater chewing enhances oral mucosal immunity.

This interpretation is broadly consistent with previous evidence that mastication can influence salivary secretion and salivary s-IgA output [5,10,11,12,14,22,23,24]. Earlier studies have mainly examined gum chewing, experimentally controlled oral stimulation, or general salivary responses to mastication [11,12,23,24]. Recent literature further supports interpreting the present findings within a broader salivary gland and oral-systemic health context. Salivary glands are essential for maintaining oral and overall health, and disease- or stimulus-related changes in salivary gland function can alter saliva composition and biological activity [25,26]. Moreover, the growing recognition of oral-systemic interactions further supports the clinical relevance of examining meal-related oral functions, such as mastication, in relation to mucosal host defense responses [27]. By contrast, the present study evaluated chewing behavior during consumption of a common food under ecologically relevant conditions and quantified chewing behavior objectively using a wearable sensor [15,16]. In this respect, the present findings extend previous work by suggesting that natural variation in chewing behavior during an ordinary meal may be associated with differences in short-term postprandial oral immune responsiveness.

A notable feature of the present findings is that the association was more apparent for pre-chewing-normalized salivary s-IgA concentration than for absolute salivary s-IgA concentration. Salivary s-IgA is known to show substantial biological variability across individuals. In the present study, baseline salivary s-IgA concentration was inversely associated with normalized postprandial responses, indicating that ratio normalization reduced but did not fully eliminate baseline-related variation. This interpretation is also consistent with the finding that the association became less pronounced after baseline adjustment. Accordingly, pre-chewing-normalized indices may be useful for descriptive evaluation of response dynamics, but they should not be interpreted in isolation from absolute values or baseline-adjusted analyses.

The findings regarding salivary s-IgA secretion rate provide an important functional perspective. Because salivary s-IgA secretion rate reflects both salivary s-IgA concentration and salivary flow, it may better represent the amount of s-IgA delivered to the oral cavity over time than concentration alone. In the present study, no significant between-group differences in salivary s-IgA secretion rate were observed at any postprandial time point. Thus, the biological implications of the normalized concentration findings should not be overstated.

The repeated-measures analysis suggested a possible time-dependent tendency, particularly at 25–30 min after eating, but the overall interaction remained borderline in the joint test. Therefore, although the data are consistent with the possibility that chewing-related differences in salivary s-IgA response may emerge over time, this pattern should still be regarded as exploratory.

The absence of significant between-group differences in saliva weight is also informative. If the greater postprandial s-IgA response observed in the ≥30 chews/bite group had simply reflected greater bulk fluid secretion, a parallel between-group difference in saliva weight would have been expected. Because this was not observed, the higher pre-chewing-normalized salivary s-IgA response in frequent chewers may not be explained solely by increased salivary output. Salivary responses during eating are influenced by multiple sensory and mechanical factors, including oral stimulation, food texture, and taste [6,13,14,22,23,24]. Repeated oral stimulation during chewing may therefore be related to the neural and glandular processes involved in salivary s-IgA secretion [5,6,14]. In this context, the present findings are consistent with, but do not establish, a possible link between masticatory behavior and short-term salivary immune responses.

The temporal pattern of the present findings also deserves attention. No significant between-group difference in pre-chewing-normalized salivary s-IgA concentration was observed at 0–5 min, whereas clear differences were present at 10–15 and 25–30 min. Similarly, the significant within-group increase in the pre-chewing-normalized s-IgA secretion rate was observed only at the later postprandial time point in the ≥30 chews/bite group. These observations suggest that the influence of chewing behavior on salivary s-IgA responses may emerge over time rather than appearing immediately after food consumption. Such a delayed response is biologically plausible, as mastication may influence the downstream neural and glandular processes that continue after eating has ceased [4,5,6,14,22]. In addition, s-IgA in saliva is generally considered to reflect the recirculation and homing of IgA-committed B cells from the broader mucosal immune system to the salivary glands over a longer time scale [28]. Therefore, the acute postprandial changes observed in the present study are more likely to reflect the neural or autonomic regulation of salivary protein secretion during eating than rapid changes in cellular homing or plasma-cell differentiation. At the same time, if the mean chewing count per bite reflects habitual chewing behavior to some extent, repeated masticatory stimulation might also be related to longer-term neuroimmune regulation of the salivary gland environment. However, the underlying mechanism was not directly examined in the present study and should therefore be interpreted cautiously. The above speculation should be regarded as exploratory and is not supported by direct immunological or autonomic measurements in the present data.

This study has several strengths. Chewing behavior was assessed objectively using a wearable sensor, saliva was sampled at multiple postprandial time points, and the test food was a rice ball consumed under familiar eating conditions in Japan [15,16]. These design features increase the practical relevance of the findings beyond highly artificial chewing tasks.

However, several limitations should also be acknowledged. First, the study population consisted only of healthy young adult men from a single institution, which limits the generalizability of the findings. Second, the sample size was modest, and the study should be interpreted as exploratory. Third, because the design was observational, causal inference is not possible. Fourth, the cut-off of 30 chews/bite was data-derived from the sample median and was used only for secondary exploratory descriptive analyses rather than as a predefined physiological target. Accordingly, the present findings should not be interpreted as evidence that 30 chews/bite represents an optimal or biologically validated threshold, although a similar chewing-count condition has been examined in previous physiological studies [21]. Fifth, although the use of a rice ball increased ecological validity, it also introduced additional sensory and behavioral influences, including food texture, taste, bolus formation, and swallowing, compared with more mechanically standardized test materials such as chewing gum or paraffin wax. Sixth, although oral health information was expanded to include present teeth, DMF teeth, CPI scores, and OHI, occlusal condition was not objectively assessed. Seventh, although all participants were non-smokers and the timing and pre-examination restrictions were standardized, other potentially relevant factors, such as psychological stress, sleep, and habitual lifestyle factors, were not directly assessed. Finally, no autonomic, endocrine, or broader immunological markers were included [5,19], and the wearable chewing sensor does not directly measure chewing force or masticatory efficiency. Therefore, measurement error cannot be completely excluded, particularly for extreme values, although the sensitivity analyses did not materially alter the overall direction of the findings. In addition, extremely high chewing counts are rarely observed during ordinary meal consumption and may reflect atypical eating behavior or limitations in bite detection by the wearable sensor. Future studies should further validate the accuracy of wearable chewing-count measurements obtained under natural eating conditions.

## 5. Conclusions

In this exploratory pilot study of healthy young adult men, greater mean chewing count per bite during rice ball consumption was associated with higher pre-chewing-normalized salivary s-IgA concentrations at 10–15 and 25–30 min after eating in nominal analyses. However, these associations were attenuated after false discovery rate correction and baseline adjustment, and no significant between-group differences were observed in salivary s-IgA secretion rate. Accordingly, the present findings should be interpreted cautiously as exploratory evidence of an association between chewing behavior and short-term postprandial salivary s-IgA responses rather than as confirmation of a biological or clinical benefit.

## Figures and Tables

**Table 1:** Pre-chewing characteristics of the participants according to mean chewing count per bite (<30 vs. ≥30 chews/bite).

Variable	<30 Chews/Bite (*n* = 18)	≥30 Chews/Bite (*n* = 19)	*p* Value
Age, years	23.0 [22.2–25.5]	25.0 [22.0–28.5]	0.350
Eating time, s	194.5 [162.0–263.0]	194.0 [170.0–230.0]	0.939
Total chewing count	237.5 [161.2–283.5]	285.0 [252.5–343.0]	0.052
Chewing tempo, chews/min	72.5 [64.5–79.0]	88.0 [83.5–92.5]	<0.001
Mean chewing count per bite	20.0 [14.5–26.0]	74.0 [44.0–121.0]	<0.001
Saliva weight before eating, g	2.4 [1.9–4.0]	2.4 [2.0–3.4]	0.616
Salivary s-IgA concentration before eating	213.5 [81.2–329.3]	157.7 [100.7–264.1]	0.820
Salivary s-IgA secretion rate before eating	77.6 [40.1–174.6]	94.4 [56.3–120.8]	0.843
Present teeth, n	28.0 [28.0–28.0]	28.0 [28.0–28.0]	0.903
DMF teeth, n	0.0 [0.0–0.0]	0.0 [0.0–1.0]	0.815
CPI periodontal pocket score ≥ 1, n (%)	5 (27.8)	3 (15.8)	0.447
CPI bleeding score ≥ 1, n (%)	3 (16.7)	2 (10.5)	0.660
OHI score	0.0 [0.0–0.1]	0.0 [0.0–0.0]	0.657

Values are presented as median [interquartile range] for continuous variables and as number (percentage) for categorical variables. Between-group comparisons were performed using the Mann–Whitney U test for continuous variables and Fisher’s exact test for categorical variables. CPI periodontal pocket and bleeding findings were defined as present when the corresponding CPI score was ≥1. The cut-off of 30 chews/bite was derived from the sample median and was used for secondary exploratory descriptive comparison. Abbreviations: s-IgA, secretory immunoglobulin A; DMF, decayed, missing, and filled; CPI, community periodontal index; OHI, oral hygiene index.

**Table 2:** Comparison of postprandial salivary s-IgA concentration, saliva weight, and salivary s-IgA secretion rate between participants in the <30 and ≥30 chews/bite groups.

Outcome	Time Point	<30 Chews/Bite (*n* = 18)	≥30 Chews/Bite (*n* = 19)	*p* Value
A. Salivary s-IgA concentration (μg/mL)				
	Before eating	213.5 [81.2–329.3]	157.7 [100.7–264.1]	0.820
	0–5 min	96.6 [49.8–141.7]	71.1 [45.1–128.7]	0.682
	10–15 min	105.2 [57.4–163.8]	152.5 [92.0–235.3]	0.118
	25–30 min	141.9 [49.7–236.8]	161.2 [93.2–268.0]	0.346
B. Pre-chewing-normalized salivary s-IgA concentration (%)				
	Before eating	100.0 [100.0–100.0]	100.0 [100.0–100.0]	1.000
	0–5 min	44.3 [37.1–70.0]	39.6 [28.4–65.2]	0.457
	10–15 min	56.2 [49.5–73.5]	74.0 [64.3–102.3]	0.005
	25–30 min	91.2 [57.0–104.5]	118.5 [90.1–139.5]	0.022
C. Saliva weight (g)				
	Before eating	2.4 [1.9–4.0]	2.4 [2.0–3.4]	0.616
	0–5 min	3.9 [2.3–5.1]	3.5 [2.9–4.2]	0.595
	10–15 min	4.3 [2.6–5.0]	4.0 [2.6–4.5]	0.412
	25–30 min	4.3 [3.3–5.2]	3.3 [2.2–4.4]	0.062
D. Pre-chewing-normalized saliva weight (%)				
	Before eating	100.0 [100.0–100.0]	100.0 [100.0–100.0]	1.000
	0–5 min	126.4 [107.5–137.8]	127.4 [112.4–171.2]	0.514
	10–15 min	134.8 [112.3–189.9]	136.9 [111.2–162.2]	0.773
	25–30 min	154.1 [117.5–223.1]	133.1 [103.8–155.3]	0.167
E. Salivary s-IgA secretion rate				
	Before eating	77.6 [40.1–174.6]	94.4 [56.3–120.8]	0.843
	0–5 min	49.5 [33.9–108.1]	54.2 [30.4–79.5]	0.727
	10–15 min	70.1 [29.7–140.9]	105.0 [54.6–151.3]	0.267
	25–30 min	106.1 [38.7–184.3]	113.5 [79.3–155.2]	0.867
F. Pre-chewing-normalized salivary s-IgA secretion rate (%)				
	Before eating	100.0 [100.0–100.0]	100.0 [100.0–100.0]	1.000
	0–5 min	59.4 [38.4–80.1]	63.2 [39.6–83.3]	0.964
	10–15 min	94.4 [56.3–110.7]	115.0 [89.4–154.9]	0.098
	25–30 min	128.9 [95.8–168.2]	135.5 [96.2–184.4]	0.750

Values are presented as median [interquartile range]. Between-group comparisons were performed using the Mann–Whitney U test. Relative values are expressed as percentages (%) with the pre-chewing value set at 100 for each participant. These group comparisons were performed as secondary exploratory descriptive analyses because the cut-off of 30 chews/bite was data-derived from the sample median. Abbreviations: s-IgA, secretory immunoglobulin A.

## Data Availability

The data supporting the findings of this study are available from the corresponding author (T.T.) on request.

## Supplementary Materials

The following supporting information can be downloaded at: https://www.mdpi.com/article/10.3390/dj14080519/s1, Table S1: Additional exploratory analyses of the association between mean chewing count per bite and postprandial salivary s-IgA response.

## Abbreviations

The following abbreviations are used in this manuscript:

s-IgA	secretory immunoglobulin A
CPI	community periodontal index
DMF	decayed, missing, and filled
FDR	false discovery rate
GEE	generalized estimating equations
IQR	interquartile range
jRCT	Japan Registry of Clinical Trials
OHI	oral hygiene index
SPSS	Statistical Package for the Social Sciences
