# Supplementary material for: Association Between Mean Chewing Count per Bite During Rice Ball Consumption and the Postprandial Salivary Secretory Immunoglobulin a Response in Healthy Adult Men: A Pilot Study
Source: Dent J (Basel). 2026 Aug 13;14(8):519. doi: 10.3390/dj14080519 (PMC13510809; doi:10.3390/dj14080519)
Supplement: Supplementary file 1 [file dentistry-14-00519-s001.zip › dentistry-4408828-supplementary.pdf]

Supplementary Table S1. Additional exploratory analyses of the association between mean chewing count per bite and postprandial salivary s-IgA response.

Analysis	Outcome	Time point	Estimate/statistic	95% CI	P value	Adjusted p or q value	Interpretation
Spearman correlation	Pre-chewing-normalized salivary s-IgA concentration	0–5 min	ρ = -0.085	—	0.618	q = 0.618	No significant association
Spearman correlation	Pre-chewing-normalized salivary s-IgA concentration	10–15 min	ρ = 0.347	—	0.036	q = 0.053	Nominal positive association, attenuated after FDR correction
Spearman correlation	Pre-chewing-normalized salivary s-IgA concentration	25–30 min	ρ = 0.352	—	0.033	q = 0.053	Nominal positive association, attenuated after FDR correction
Baseline–normalized association	Baseline salivary s-IgA concentration vs. pre-chewing-normalized salivary s-IgA	0–5 min	ρ = -0.374	—	0.023	—	Inverse association, indicating residual baseline-related variation

	concentration						
Baseline–normalized association	Baseline salivary s-IgA concentration vs. pre-chewing-normalized salivary s-IgA concentration	10–15 min	$\rho = -0.337$	—	0.041	—	Inverse association, indicating residual baseline-related variation
Baseline–normalized association	Baseline salivary s-IgA concentration vs. pre-chewing-normalized salivary s-IgA concentration	25–30 min	$\rho = -0.278$	—	0.095	—	Similar tendency, not statistically significant
Baseline-adjusted analysis	Log-transformed postprandial salivary s-IgA concentration	0–5 min	$\beta = -0.0010$	-0.0048 to 0.0028	0.597	—	No significant association after adjustment
Baseline-adjusted analysis	Log-transformed postprandial salivary s-IgA	10–15 min	$\beta = 0.0028$	-0.0014 to	0.184	—	Positive but not statistically significant

	concentration			0.0070			after adjustment
Baseline-adjusted analysis	Log-transformed postprandial salivary s-IgA concentration	25–30 min	$\beta = 0.0032$	-0.0017 to 0.0081	0.192	—	Positive but not statistically significant after adjustment
GEE analysis	Pre-chewing-normalized salivary s-IgA concentration: main effect of mean chewing count per bite	Overall	$\beta = -0.032$	−0.171 to 0.107	0.649	—	No overall main effect
GEE analysis	Mean chewing count per bite × time interaction	10–15 min	$\beta = 0.189$	−0.018 to 0.396	0.074	—	Borderline positive interaction
GEE analysis	Mean chewing count per bite × time interaction	25–30 min	$\beta = 0.259$	0.021 to 0.498	0.033	—	Positive interaction at 25–30 min
GEE analysis	Joint Wald test for interaction terms	Overall	$\chi^2 = 5.718$	—	0.057	—	Overall interaction borderline, not

							conventionally significant
Sensitivity analysis excluding participants with >100 chews/bite	Pre-chewing-normalized salivary s-IgA concentration	0–5 min	ρ = -0.137	—	0.471	q = 0.471	No significant association
Sensitivity analysis excluding participants with >100 chews/bite	Pre-chewing-normalized salivary s-IgA concentration	10–15 min	ρ = 0.243	—	0.196	q = 0.294	Positive direction preserved, statistical evidence weaker
Sensitivity analysis excluding participants with >100 chews/bite	Pre-chewing-normalized salivary s-IgA concentration	25–30 min	ρ = 0.429	—	0.018	q = 0.054	Positive association preserved, borderline after FDR correction
Sensitivity analysis excluding the Tukey outlier	Pre-chewing-normalized salivary s-IgA concentration	0–5 min	ρ = -0.063	—	0.716	q = 0.716	No significant association
Sensitivity analysis excluding the Tukey outlier	Pre-chewing-normalized salivary s-IgA	10–15 min	ρ = 0.357	—	0.032	q = 0.049	Positive association preserved and FDR-significant

	concentration						
Sensitivity analysis excluding the Tukey outlier	Pre-chewing-normalized salivary s-IgA concentration	25–30 min	ρ = 0.418	—	0.011	q = 0.034	Positive association preserved and FDR-significant

Spearman correlations were evaluated between mean chewing count per bite and pre-chewing-normalized salivary s-IgA concentration at each postprandial time point. False discovery rate (FDR) correction was applied across the three postprandial time points using the Benjamini–Hochberg procedure. Baseline-adjusted analyses used log-transformed postprandial salivary s-IgA concentration as the dependent variable and included baseline salivary s-IgA concentration as a covariate. Generalized estimating equations (GEE) were used to account for repeated measures over time. Sensitivity analyses were performed after excluding participants with mean chewing counts exceeding 100 chews/bite and after excluding the statistical outlier identified using Tukey's method. Abbreviations: s-IgA, secretory immunoglobulin A; GEE, generalized estimating equations; CI, confidence interval; FDR, false discovery rate.
